# The complex rupture evolution of the long and slow, tsunamigenic 2021 South Sandwich Islands earthquake

**DOI:** 10.1038/s41598-025-02043-6

**Published:** 2025-05-21

**Authors:** Ryo Yamaguchi, Yuji Yagi, Ryo Okuwaki, Bogdan Enescu

**Affiliations:** 1 Graduate School of Science and Technology, University of Tsukuba, Tennodai 1-1-1, Tsukuba, 305–8572 Ibaraki Japan; 2 Institute of Life and Environmental Sciences, University of Tsukuba, Tennodai 1-1-1, Tsukuba, 305–8572 Ibaraki Japan; 3 Department of Geophysics, Graduate School of Science, Kyoto University, Kitashirakawa, Oiwake-cho, Sakyo-ku, Kyoto, 606-8502 Japan; 4 National Institute for Earth Physics, Calugareni str. 12, P.O. Box MG-2, Magurele-Bucharest, Ilfov, 077125 Romania

**Keywords:** Seismology, Tectonics

## Abstract

On August 21, 2021, a large earthquake occurred in the South Sandwich subduction zone, and the associated tsunami was widely observed. To robustly analyse the detailed seismic source process of this long-source duration (over 200 s) event occurring in a convexly shaped subduction zone, we applied the Potency Density Tensor Inversion with a non-rectangular and non-planar source surface to the broadband teleseismic *P*-waves. This method allows us to suitably reduce the effect of the time-increasing uncertainty of the Green’s function and the effect of modelling errors related to the fault geometry. We found the slip vectors of the 400 km long rupture area rotate clockwise to the south, corresponding to the clockwise rotation of the trench strike. Our results reveal that the rupture propagated up-dip to the shallow region, then propagated to the south-southeast along the trench, and stagnated for about 30 s at around 130 km south-southeast from the epicentre. After the stagnation of the rupture front, the moment-rate gradually increased with time, although a clear rupture area could not be identified for about 45 s. Afterwards the rupture re-propagated south-southwest along the trench from the stagnation area. The slow rupture growth following the stagnation of the fast rupture triggered a new fast rupture, which led to the 2021 South Sandwich Islands earthquake having the typical characteristics of a tsunami earthquake, with a long rupture duration and a slow average rupture front velocity.

**Supplementary Information:**

The online version contains supplementary material available at 10.1038/s41598-025-02043-6.

## Introduction

Understanding the rupture process of tsunami-generating earthquakes is important for assessing the potential for future tsunami events. A “tsunami earthquake” is an event which has rupture characteristics that lead to the generation of tsunamis that are unusually large given the event’s magnitude^1,2^. It has been pointed out that tsunami earthquakes have a slow rupture front velocity and long rupture duration^3,4^. In the case of the 1992 Nicaragua earthquake, one of the typical tsunami earthquakes, the rupture front velocity and the rupture duration were reported to be 1.5 km/s, or less, and ~ 110 s, respectively^2^; it has been suggested that the slow slip along the plate interface occurred due to accumulated soft subducted sediments^2^. The mechanism of tsunami earthquakes is disputed in the scientific literature: variable frictional properties on the plate interface^5^, off-fault rupture^6^, accretionary prism rupture caused by rapid stress loading^7^, thermal pressurization^8^ and so on have been suggested being responsible for the heterogeneous rupture behaviour, typically characterized by a slow and long rupture process.

**Fig. 1:**
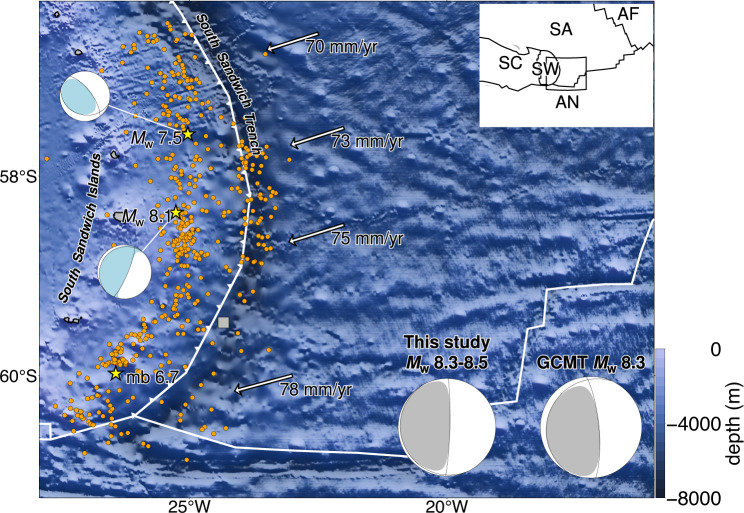
Summary of the study region. Three yellow stars are the epicentres of *M*_W_ 7.5, *M*_W_ 8.1 and mb 6.7 events reported by the USGS NEIC^17^, and two light blue beachballs are the moment tensors of *M*_W_ 7.5 and *M*_W_ 8.1 events. The grey square is the centroid location reported by the Global Centroid Moment Tensor (GCMT) project^18,19^, and the grey beachballs on the bottom right corner are the GCMT solution and the total moment tensor solution obtained in this study. The orange dots are the aftershocks from 2021-08-12 18:32:52 to 2021-8-15 18:32:52 (within three days after the *M*_W_ 7.5 events) detected by the USGS NEIC. The vector shows the subduction velocity and azimuth of South American plate^14^. The white line indicates the plate boundary^15^, and the white front is the South Sandwich trench. The shaded background bathymetry is derived from SYNBATH^16^. The inset map shows the plates around the study region: Sandwich (SW), Scotia (SC), South American (SA), Antarctic (AN) and Africa (AF) plates^15^. The black lines are the plate boundaries^15^ and the box outlines the extent of this figure.

On August 12, 2021, the 2021 South Sandwich Islands (SSI) earthquake struck offshore the South Sandwich Islands, in the shallow part of the South Sandwich subduction zone^9,10^ classified as tectonic-erosion type^11–13^. In this subduction zone, the South American plate is subducting under the Sandwich plate at a rate of 70–78 mm/yr, in a west-southwest direction^14^ (Fig. 1). The South Sandwich trench curves convexly to the east^15^ (Fig. 1), and the South American plate is subducting obliquely in the southern region of this subduction zone. The bathymetric data on the South American plate^16^ shows several chains of seamounts extending in east-southeast to west-northwest directions. The aftershock distribution determined by the U.S. Geological Survey (USGS) extends from ~ 100 km north to ~ 300 km south of the mainshock epicentre along the South Sandwich trench^17^.

The USGS identified three independent events corresponding to the 2021 SSI earthquake (Fig. 1). At 18:32:52 (UTC), a moment magnitude (*M*_W_) 7.5 event occurred at 57.567° S, 25.032° W was followed 145 s later by an *M*_W_ 8.1 event occurred about 90 km south of the epicentre of the first event; finally, 102 s later, a body-wave magnitude (mb) 6.7 event occurred about 280 km south-southeast (SSE) of the epicentre of the first event (Fig. 1). The Global Centroid Moment Tensor (GCMT) project^18,19^ reported only a *M*_W_ 8.3 earthquake with a centroid at 59.48° S, 24.34° W (Fig. 1). Jia et al. (2022)^9^ applied the multiple point source inversion to the 2021 SSI earthquake using the low-frequency waveforms (0.002–0.05 Hz) and identified five sub-events: two thrust sub-events in the first 50 s, one long-period thrust sub-event for about 180 s with a centroid time of 90 s after the beginning of the rupture, and two thrust sub-events which occurred 3 min after the beginning of the rupture. Metz et al. (2022)^10^ carried out moment tensor inversion and finite fault inversion (FFI), and reported four sub-events. They also performed a teleseismic back-projection (BP) analysis and reported high-frequency (0.5–2 Hz) energy emitters migrating southward. Previous studies used long-period (20 s and longer) waveforms to perform source inversion and have shown that the 2021 SSI earthquake is composed of multiple sub-events^9,10^ and has the characteristics of a typical tsunami earthquake, with slow rupture front velocity and long rupture duration^9^. On the other hand, it also involves spatiotemporally complex high-frequency rupture inferred from the BP analysis^10^. To understand the rupture process of the 2011 SSI earthquake, which has the characteristics of a tsunami earthquake, it is essential to construct a detailed seismic source model that is able to explain the broadband teleseismic body waves.

In general, it is difficult to stably estimate the detailed seismic source process of an earthquake with complex fault geometries and a long rupture duration, such as the 2021 SSI earthquake. The influence of the uncertainty of the Green’s functions becomes dominant as the source duration gets longer^20^. A planar rectangle fault plane that is often adopted in the FFI may not necessarily be suitable for the actual curved and convex source fault, which can also increase the modelling uncertainty^21,22^. The recently proposed Potency Density Tensor Inversion (PDTI) has made it possible to estimate detailed source processes, including information on focal mechanism, by reducing the effects of the uncertainty of the Green’s functions^21,23^. In this study we apply the PDTI with a non-rectangular and non-planar source surface to the observed teleseismic waveforms of the 2021 SSI earthquake to estimate its source process, including spatiotemporal changes in fault geometry and slip vector. We discuss the complex rupture propagation during the 2021 SSI earthquake and propose the possibility of partial slip partitioning.

## Method

In general, the earthquake source can be expressed by the volume density of the moment-rate tensor^24^. The moment-rate volume-density tensor is calculated by multiplying the potency-rate volume-density tensors by the rigidity. In the case of the fault dislocation, the potency-rate tensor is represented by a linear combination of five basis double-couple components^21,25^. Therefore, the teleseismic waveform of an earthquake observed at station $\:j$ is given by:

1 $$\:\begin{array}{c}{u}_{j}\left(t\right)=\sum\limits_{q=1}^{5}{\int\:}_{V}^{}{\dot{P}}_{q}\left(\xi\:,t\right)*{G}_{qj}\left(\xi\:,t\right)\:dV+{e}_{bj}\left(t\right)\end{array}$$

where *V* is the 3-D source area, $\:{\dot{P}}_{q}$ is a potency-rate volume-density function of $\:q$th basis double-couple component, $\:{G}_{qj}$ is a true Green’s function of the $\:q$th basis double-couple component, $\:\xi\:$ is the location in the *V*, $\:{e}_{bj}$ is the background and instrumental noise and $\:*$ denotes the convolution operator in the time domain.

In the FFI of seismic waveforms, the potency volume-density tensor is approximated by the fault slip vector (potency areal-density vector) on a pre-assumed fault plane^26,27^. The FFI method can resolve the spatiotemporal distribution of the potency density-rate (slip-rate) on the fixed fault plane, however it cannot identify fault ruptures with a different fault geometry.

In the multiple point source inversion, the moment volume-density tensor is approximated by a sum of the moment tensors at multiple point sources^25,28,29^. This approach makes no assumptions about the fault plane and thus allows discussion of the possibility of slip occurring on an unknown fault. However, the multiple point source inversion method is unable to estimate the detailed rupture process for each sub-event. In addition, only long-period waves can be analysed due to the effect of the modelling errors caused by the simplification of the source model.

In the PDTI method, the potency-rate volume-density tensor is approximated by the potency-rate areal-density tensor on a pre-assumed model surface^21^. Then, Eq. (1) becomes

2 $$\:\begin{array}{c}{u}_{j}\left(t\right)\approx\:\sum\limits_{q=1}^{5}{\int\:}_{S}^{}{\dot{P}{'}}_{q}\left(\xi\:,t\right)*{G}_{qj}\left(\xi\:,t\right)dS+{e}_{bj}\left(t\right)\end{array}$$

where $\:S$ is 2-D model surface and $\:{\dot{P}{'}}_{q}$ is a potency-rate areal-density function. As a result, the ruptures occurring on various faults are projected onto the model surface as the potency-rate areal-density tensor. In the FFI method, the number of degrees of freedom of the potency tensor is reduced from five to two by specifying the fault plane^26,27^. However, in the PDTI method, the number of degrees of freedom of the potency tensor remains at five because the fault plane is never specified^21^. In other words, by increasing the degrees of freedom of the model, PDTI is capable of estimating a detailed seismic source process model including information on the fault geometry.

In general, a high degree of model freedom can lead to problems such as overfitting and unstable solutions. To avoid these problems, the PDTI method incorporates the modelling error derived from the uncertainty of the Green’s function into the data covariance matrix, as proposed by Yagi and Fukahata (2011)^20^, and applies Akaike’s Bayesian Information Criterion (ABIC)^30–32^ to reasonably evaluate the strength of the smoothing constraint. In this study, we use the latest version of PDTI, which introduces a time-adaptive smoothing constraint^23^ to avoid the problem of over-smoothing caused by fixing the smoothing strength.

For Eq. (2) to work successfully, it is necessary to reduce the modelling error by making the model surface $\:S$ closer to the earthquake source faults. In this study, we analysed the source process by projecting the potency density tensor distribution onto a non-planar model created by referring to the slab geometry data in Slab2.0^33^.

### Data and model parameterization

**Fig. 2:**
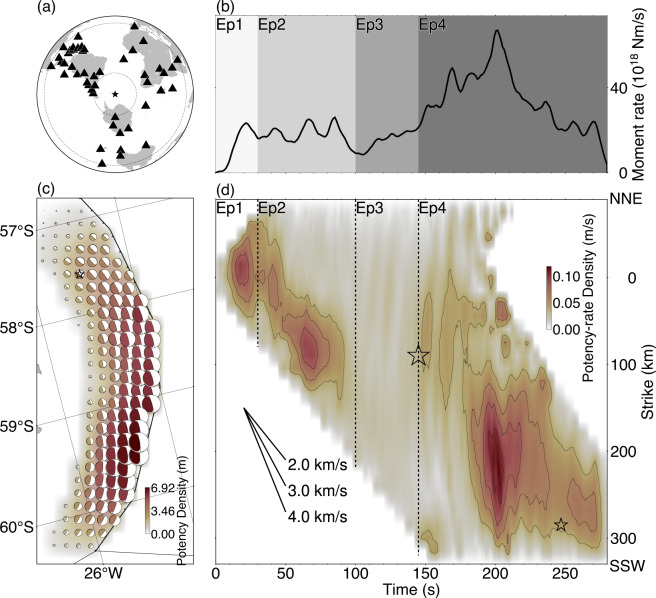
Summary of inversion results. **(a)** The station distribution used in the inversion, projected on the azimuthal equidistant map. The dotted circles show epicentral distances of 30° and 100°. **(b)** Moment rate function. The background grey colours show the time range of four episodes defined in this study. **(c)** Map projection of the potency density tensor distribution on the non-planar model. The star and black line indicate the epicenter^17^ and plate boundary^15^, respectively. **(d)** Potency-rate density evolution projected along strike. The contours of 0.04, 0.06, 0.08 and 0.10 m/s are shown as black lines. The vertical axis is distance from the epicentre. The large and small stars are the epicentres of the *M*_W_ 8.1 and mb 6.7 events detected by the USGS^17^.

**Fig. 3:**
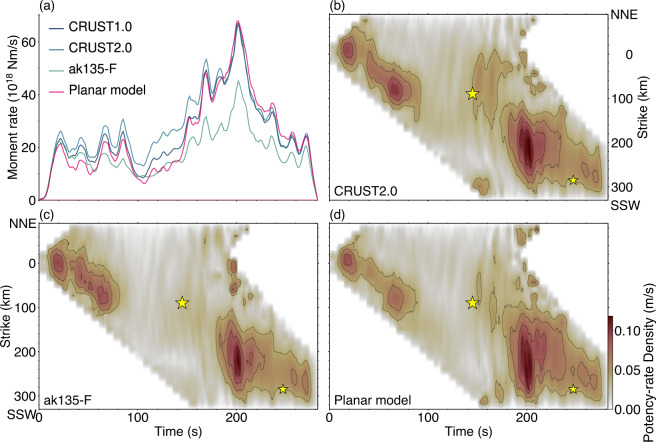
Summary of results from model sensitivity test. **(a)** The moment-rate functions when the Green’s function is calculated using two different structure models (CRUST2.0^35^ and ak135-F^36,37^; see Supplementary Tables S2 and S3) and when the potency-rate density tensor distribution is projected onto a planar model with a strike of 190° and dip of 10°. **(b, c)** Potency-rate density evolution obtained with CRUST2.0^35^ (see Supplementary Table S2) and ak135-F^36,37^ (see Supplementary Table S3) structure models for calculating the Green’s function, respectively. **(d)** Potency-rate density evolution obtained using the planar model. The large and small stars plotted in each figure are the epicentres of the *M*_W_ 8.1 and mb 6.7 events, respectively detected by the USGS^17^.

For the PDTI, we used the vertical components of the teleseismic body *P-*waves from 47 stations at epicentral distances of 30°–100° downloaded from the SAGE Data Management Center (Fig. 2a). We selected stations, ensuring a high signal-to-noise ratio and better azimuthal coverage (Fig. 2a). We converted the observed waveforms to velocity waveforms by removing the instrument response and then decimated the signal by using a 1.2 s sampling. We calculated the theoretical Green’s functions using the method of Kikuchi and Kanamori^25^ with a sampling rate of 0.1 s. We used the 1-D averaged structure of the CRUST1.0^34^ in the source region to calculate the theoretical Green’s function (see Supplementary Table S1). Other structure models, CRUST2.0^35^ (see Supplementary Table S2) and ak135-F spherical average model^36,37^ (ak135-F) (see Supplementary Table S3), are also used to evaluate the robustness of the modelling (Fig. 3).

According to the USGS aftershock distribution^17^, the 2021 SSI earthquake is assumed to have occurred near the subducted and curved slab surface. To cover the aftershock distribution within three days of the event (Fig. 1), we followed Yamashita et al. (2021)^38^ and set up a non-rectangular model plane with the strike and dip angles of 190° and 10°, respectively, with a maximum width of 150 km and length of 405 km, which is expanded using a bilinear B-spline with a knot spacing of 15 km in both the strike and dip directions. Then the depth of each knot is adjusted based on Slab2.0^33^ to minimize the location error in the Green’s function. We adopted a hypocentre (initial rupture point) by using the USGS-determined epicentre^17^ of the first event (57.567° S, 25.032° W) and the corresponding depth of the Slab2.0^33^ at 21.6 km. To enable fast rupture propagation immediately after an initial break and to reduce the number of model parameters, a hypothetical rupture initiation time was set for each spatial node. The hypocentre and the surrounding nodes were set to be able to rupture immediately after the initial break, and the other nodes were set to be able to rupture immediately after the hypothetical 2.0 km/s front had passed. Based on the reported long duration of the 2021 SSI earthquake^9,10^, the rupture duration at each knot is 180 s with a sampling interval of 1.2 s, and the total rupture duration is 280 s.

In general, the PDTI method adjusts the waveform length to mitigate the influence of the *PP-*waves on the inversion results. However, when this scheme is applied to an earthquake with a long rupture duration, such as the 2021 SSI earthquake, only a few observation points contribute to the results after 200 s from the origin time. The source time function of the 2021 SSI earthquake obtained using the empirical Green’s function^39,40^ shows that the peak value of the moment rate in the 80 s after the origin time is sufficiently smaller than the peak value in the 200 s after the origin time (see Supplementary Fig. S1). Therefore, in this study, the waveform from the arrival of the *P-*wave to 80 s after the arrival of the *PP-*wave was used for inversion to stabilize the solution after 200 s from the origin time.

## Results

**Fig. 4:**
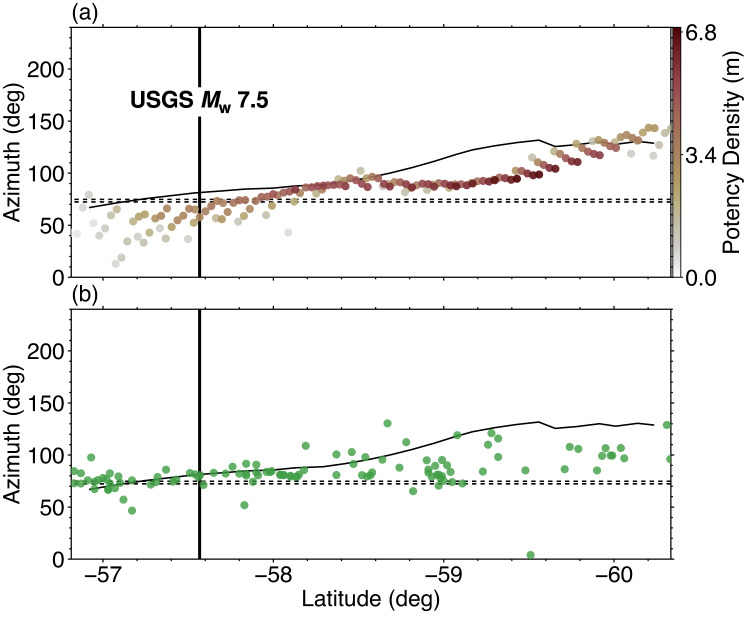
Spatial variation of slip vectors. **(a)** The spatial variation of total slip vectors for each knot obtained by PDTI. The slip vectors are calculated for the preferred nodal plane. **(b)** The spatial variation of slip vectors for GCMT solutions^18,19^ of background events larger than *M*_W_ 5.5. In both **(a)** and **(b)**, the black solid line is the direction perpendicular to the strike of the subducting slab near the trench. The two black dashed lines indicate the direction of plate subduction^14^, the upper line and the lower line are corresponding to the most northerly and to the most southerly subduction vector azimuths denoted as arrows in Fig. 1, respectively. The vertical line indicates the initial rupture point.

The spatial distribution of the potency density tensor, calculated by taking the temporal integration of potency-rate density functions at each knot, is dominated by thrust focal mechanisms that are similar to the total moment tensor (Figs. 1 and 2c). The source area spans about 400 km in length, with a maximum potency of 6.4 m at about 210 km south-southeast of the epicentre (Fig. 2c). The estimated total moment tensor solution, obtained by the spatiotemporal integration of the potency-rate density tensors, shows a thrust focal mechanism including a 25% non-double-couple component (Fig. 1). The total seismic moment is 6.47 × 10^21^ N m (*M*_W_ 8.5). The slip vector of the nodal plane closest to the slab surface geometry shows a tendency to rotate clockwise to the south-southwest (SSW) (Fig. 4a). The moment rate function has ten or more spikes with the largest peak of 6.71 × 10^19^ N m/s at 201 s (Fig. 2b). The synthetic waveforms calculated from the obtained source process model well reproduce the observed waveforms, including the data points not used for the inversion (Supplementary Fig. S2). In this study, we define Episodes 1–4 based on the moment rate function (Fig. 2d) and snapshots (Supplementary Figs. S3, S4, S5).

**Fig. 5:**
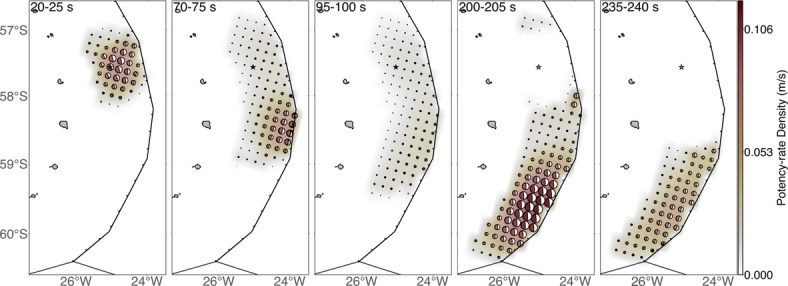
Selected snapshots of the potency-rate density distribution (Fig. 2d). The beachballs show the potency-rate density tensors. The star and black line indicate the epicentre^17^ and plate boundary^15^, respectively.

In Episode 1, the thrust-type rupture propagated mainly on the up-dip side of the hypocentre and then reached the trench at 30 s after the origin time (OT) (Fig. 5, Supplementary Fig. S3). Figure 2d shows the time evolution of the rupture area projected along the strike of the model plane (190°). As shown in Fig. 2d, the thrust-type rupture also propagated asymmetrically in the SSW and north-northeast (NNE) directions until OT + 30 s. The rupture front velocity in the strike direction is about 2 km/s. The SSW rupture started at OT + 8 s, and the rupture front velocity in the strike direction is about 3 km/s.

In Episode 2, from OT + 30 s, the thrust-type rupture propagated unilaterally in southward direction along the trench, at the rupture front velocity in the strike direction of about 3 km/s from OT + 30 s to OT + 70 s and then the southward rupture propagation stagnated at around 130 km SSW from the epicentre (Figs. 2d and 5, Supplementary Fig. S3). Within this episode, a large fault slip event, with a peak along the trench at about 130 km SSE of the epicentre, begins at OT + 55 s and continues for about 35 s.

In Episode 3, the moment-rate function increases gradually from about OT + 100 s (Fig. 2d). This gradual increase continues for about 45 s. We note it is difficult to identify a clear rupture propagation during Episode 3 due to the relatively small potency-rate density (Fig. 2d, Supplementary Fig. S4).

Episode 4 begins with the rapid rise in the moment-rate function around OT + 145 s (Fig. 2d). This initiation coincides with the origin time of the secondary *M*_W_ 8.1 event, as determined by the USGS (Fig. 2d). The thrust-type rupture propagated in the SW direction from around 100 km SSW of the epicentre and reached the southern end of the model area at about OT + 200 s (Fig. 2d, Supplementary Fig. S5). The maximum peak of the moment rate function equals 6.71 × 10^19^ N m/s, at 202 s (Fig. 2d).

## Model sensitivity tests

In this study, we calculated the Green’s functions using the 1-D structure model that averaged the CRUST1.0^30^ structure in the source region. In general, the longer the rupture duration, the more likely it is to be affected by the uncertainty of the Green’s functions^20^. The effect of the Green’s function uncertainty is introduced to the data covariance matrix according to Yagi and Fukahata (2011)^20^, but this approach cannot evaluate the effect of non-Gaussian errors originating from the model setting^41^. Although the effect of non-Gaussian errors originating from the setting of the structure model can be evaluated using a Bayesian multi-model estimation^41–43^, it is not practical to apply this approach to PDTI in terms of computational cost^44^. Therefore, we examined the behaviour of the solutions for two additional structure models: CRUST2.0^35^ and ak135-F^36,37^. In addition to the structure sensitivity test, we also examined the behaviour of the solution when the model surface was set to a plane with a strike of 190° and dip of 10°, rather than the non-planar surface referenced in Slab2.0^33^. In this test, a 1-D structure that averaged the CRUST1.0 model in the source region is used.

Figure 3 shows the variation in the solution when the structure or depth of the model surface is perturbed. The timing of the peaks of the moment-rate function, which can be seen in Episode 1, 2 and 4, is slightly perturbed depending on the model, but similar results are obtained for all models (Fig. 3a). This result is consistent with the results obtained by the model sensitivity test in previous research^21^. On the other hand, the amplitude of the moment-rate function is almost the same for all models in Episode 1, but the model dependence of amplitude increases after Episode 2 (Fig. 3a). This result suggests that the effect of model uncertainty increases over time. However, the key features of the moment-rate function, such as the gradual increase in the moment-rate during Episode 3 and the rapid increase in the moment-rate at the start of Episode 4, are reproduced in all models (Fig. 3a). The asymmetric bilateral rupture propagating in the NNE–SSW direction in Episode 1, the unidirectional rupture propagating in the SSW direction in Episode 2, the absence of a clear event in Episode 3, and the large slip event occurring 200 km SSW from the hypocentre in Episode 4 are all reproduced in the three alternative models (Fig. 3b, c, d). The rupture area at the start of Episode 4 differs depending on the model, but a relatively large potency-rate density region near the epicentre of the USGS’ secondary *M*_W_ 8.1 event is commonly obtained in all three alternative models (Fig. 3b, c, d). The moment magnitudes are perturbed by the model setting, and the values range from *M*_W_ 8.3–8.5 when using the different structural models and model geometries.

## Discussion

We analysed the broadband teleseismic body *P-*waves from the 2021 SSI earthquake and found that the rupture process can be divided into four episodes (Fig. 2b, d). In Episode 1, the rupture propagated from the hypocentre to the shallow region while expanding in the NNE–SSW direction, reaching the South Sandwich trench (Supplementary Fig. S3). In Episode 2, the rupture propagated in the SSE direction along the trench, but after OT + 70 s, it remained stagnant around 130 km SSE of the epicentre (Supplementary Fig. S3). In Episode 3, a clear rupture area cannot be identified (Supplementary Fig. S4), but the moment-rate increases gradually with time (Fig. 2b). In Episode 4, the rupture propagated towards SSW, until OT + 200 s (Supplementary Fig. S5). Our seismic source model explains the broadband teleseismic body *P-*waveforms, and its characteristics are reproduced even when other three different model settings are used (Fig. 3). Evaluating the smoothing strength using ABIC^30–32^ prevents overfitting and makes it possible to estimate robust results. It is worth noting that the PDTI results are smoothed according to the amount of information in the data. In the following, we discuss a series of fast and slow rupture evolution in particular comparing with the previous BP analysis^10^, exhibiting the typical tsunami-earthquake characteristics but having a more heterogeneous rupture evolution. We also discuss a possibility of slip partitioning based on our finding of rotation of slip vector azimuths.

The PDTI method employed in this study reduces the effects of modelling errors by increasing the number of degrees of freedom of fault geometry and rupture front^21,45,46^, while the BP method resolves the rupture propagation process by tracking the wave radiation sources without requiring detailed model setting^47–49^. However, the BP that uses the standard formulation may not be capable of robustly estimating the source radiation, partly because (as revealed by Jia et al. (2022)^9^ and our PDTI result in this study) the 2021 SSI earthquake involves the focal mechanism change during the rupture process and it violates the implicit assumption of the standard BP formulation (e.g., a single radiation pattern throughout the rupture process). Moreover, careful selection of the regional arrays and travel-time calibration should be required to better perform the BP for this earthquake^10^, so here we compare our results with the BP result of Metz et al. (2022)^10^. The results of the BP method using high-frequency waveforms^10^ show that the rupture propagated in the SSE direction from the epicentre until OT + 100 s. The BP signal remained at a low level from OT + 100 to 160 s, while the strong BP signals are distributed about 250 km south of the epicentre after OT + 200 s^10^. Considering that the high-frequency waveforms are generally sensitive to disturbances of slip-rate and the rupture front propagation^50,51^, the results of this study can be compared with the BP result. The SSE rupture propagation until OT + 100 s obtained by the BP method corresponds to Episodes 1 and 2 of this study, the low-level BP signal from OT + 100 to OT + 145 s corresponds to Episode 3, and the strong BP signal from OT + 200 s corresponds to the large rupture event of Episode 4. After the start of Episode 4, the BP signal increases, but it is weaker than the other major BP signals^10^. This may suggest that the initial rupture of Episode 4 accelerated smoothly.

The averaged rupture front velocity of the 2021 SSI earthquake is about 1.5 km/s, estimated from the distance and time difference from the initial break and the major rupture event of Episode 4 (Fig. 2d), in agreement with previous research^9,10^. This slow rupture front velocity appears to reflect the characteristics of a tsunami earthquake^2,52^. However, our results also show that the rupture front propagated relatively fast in Episodes 1, 2 and 4. In Episode 1, the rupture front velocity in the strike direction is about 2–3 km/s (Fig. 2d). Considering that the rupture is not only propagating in the strike direction, but also towards the shallow region, the actual rupture front velocity may reach about 2.8–4.2 km/s. In Episode 2, the rupture front velocity in the strike direction is about 3 km/s (Fig. 2d). Adjusting for discrepancy between the strike direction and the actual rupture propagation direction, the rupture front velocity in Episode 2 is about 3.2 km/s. In the latter half of Episode 2, the rupture stagnates in the area around 59°S. During this stage, we observe the variation of the dip angle in our potency-rate density tensors; the higher dip angles, in particular, show variation in the shallow domain (down to ~ 10 km depth) (Fig. S7). A similar but shorter stagnation for about 10 s has been observed for the 2010 El Mayor-Cucapah earthquake, which is attributed to the geometric complexity of the fault^53^. Around the source region of the 2021 SSI earthquake, the convex structure that may be associated with subducting fracture zones and/or seamounts is observed on the oceanic plate near 59°S^16^, which can induce strength heterogeneity and affect rupture propagation. The smoothing strength increases over time because of the increase with time of the uncertainty of the Green’s function, thus it is difficult to trace the rupture front in Episode 4. If we take the USGS hypocentre of the *M*_W_ 8.1 event as the starting point of Episode 4, the rupture velocity in Episode 4 is about 3.2 km/s (Fig. 2d). The rupture front velocity that can be identified separately for Episodes 1,2 and 4 is about 60–90% of the S-wave velocity, which is consistent with the rupture front velocity of regular earthquakes^54,55^.

In Episode 3, the moment-rate increases gradually for about 45 s, but a clear rupture area cannot be identified (Fig. 2b, d). On the other hand, the BP analysis results show that the source of the wave corresponding to Episode 3 is stagnating at a point about 110 km south-southeast of the hypocentre^10^. The BP analysis results suggest that the rupture area of Episode 3 is distributed around the rupture stagnation area of Episode 2 and the rupture initiation area of Episode 4. The gradual increase in the moment rate reflects the gradual increase in the rupture area and/or the gradual acceleration of fault slip-rate. Therefore, the shear stress loading due to the gradual expansion of the rupture area and/or the gradual acceleration of slip-rate may trigger the rupture of Episode 4. Notably, the long-period, slow rupture event has been reported by previous studies^9,10^; Jia et al.^9^ determined the long-period event with a peak at around 100 s in the shallow zone, based on analysis of the low-frequency components. We thus consider that the gradual increase of moment rate with less obvious rupture migration seen during Episode 3 (slow rupture growth) is relevant to the long-period event, and our source model is integrating those diverse source signatures obtained by previous studies (Supplementary Fig. S6).

In oblique subduction zones, slip partitioning often occurs between dip-slip interplate faults and strike-slip faults in the continental crust^56,57^. When slip partitioning occurs, the slip vector of the dip-slip fault can point in a direction between the relative plate subduction direction and the direction normal to the trench axis^56,58,59^. Figure 4a shows the spatial variation of the slip vector of the nodal plane closest to the slab surface geometry. The slip vectors obtained in this study show a tendency to rotate clockwise as the slip propagates south-southwest in response to the change in the trench strike. In the rupture region spanning the latitudes 57°S–58.4°S, the slip vector azimuth becomes larger towards the south, while at latitudes 58°S–58.4°S it becomes consistent with the trench-normal direction (Fig. 4a). Between latitudes 58.4°S–59.3°S, the slip vector azimuth is about 15° greater than the direction of plate subduction, and deviates from the trench-normal direction (Fig. 4a). From 59.3°S to 60.2°S, the slip vector azimuth becomes larger towards the south, and from 59.8°S–60.2°S it approaches the trench-normal direction again (Fig. 4a). A similar result can be observed using the background seismicity (earthquakes of *M*_W_ > 5.5) from the GCMT solutions: the dip-slip vector azimuths seem to align with the trench-normal direction at latitudes 57°S–58°S, while the azimuths tend to orient between the trench-normal direction and subduction direction at latitudes 58°S–60°S (Fig. 4b). The spatial variation of the dip-slip vectors, including our results, suggests that slip partitioning^56,58,59^ may play a role in the convex South Sandwich subduction zone.

## Conclusion

We estimated the spatiotemporal evolution of rupture propagation during the 2021 South Sandwich Islands earthquake by using the PDTI method. The results of the model sensitivity test using the three alternative models suggest that the rupture propagation process obtained in this study is stable. The rupture can be divided into four episodes. In Episode 1 (0–30 s), the fast rupture propagated in a shallow direction, in Episode 2 (35–100 s) the fast rupture propagated southeast along the trench and then stagnates, in Episode 3 (100–145 s) the rupture slowly expanded around the stagnant area, and in Episode 4 (145–280 s), the fast rupture propagated in a south-southwest direction along the trench. The results of this study demonstrate that the characteristics of tsunami earthquakes, such as long rupture duration and a slow average rupture front velocity, are observed in the case of the 2021 South Sandwich Islands earthquakes as a complex process: slow rupture growth after stagnation of the initial fast rupture and the triggering of a final fast rupture. The spatial variation of the slip vectors shows a tendency to rotate clockwise to the south, corresponding to the clockwise rotation of the strike of the convex South Sandwich trench to the south. Our detailed source model successfully explains the broadband tele-seismic *P-*waves and can provide a unified explanation of the previous back-projection result and the multiple point source inversion results of the long-period waveforms, shedding light on the highly heterogeneous rupture process of the 2021 South Sandwich Islands earthquake.

## Electronic supplementary material

Below is the link to the electronic supplementary material.

Supplementary Material 1

## Data Availability

All seismic data were downloaded from IRIS Wilber 3 system (https://ds.iris.edu/wilber3/) or IRIS Web Services (https://service.iris.edu/), including the following station networks: (1) Caribbean Network (CU; https://doi.org/10.7914/SN/CU); (2) GEOSCOPE (G; https://doi.org/10.18715/GEOSCOPE.G); (3) the Global Telemetered Seismograph Network (USAF/USGS) (GTSN) (GT; https://doi.org/10.7914/SN/GT); (4) the IRIS/IDA Seismic Network (II; https://doi.org/10.7914/SN/II) and (5) the Global Seismograph Network (IU; https://doi.org/10.7914/SN/IU). The moment tensor solutions of the Global Centroid Moment Tensor (GCMT) catalog are available through the website https://www.globalcmt.org/CMTsearch.html. The CRUST1.0, CRUST2.0 and ak135-F are available through the websites https://igppweb.ucsd.edu/~gabi/crust1.html, https://igppweb.ucsd.edu/~gabi/crust2.html and http://rses.anu.edu.au/seismology/ak135/ak135f.html, respectively. The Slab2 model is available at https://doi.org/10.5066/F7PV6 JNV. Plate motion data are obtained from Plate Motion Calculator (http://ofgs.aori.u-tokyo.ac.jp/~okino/platecalc_new.html). We used Generic Mapping Tools (v6.5.0) (https://docs.generic-mapping-tools.org/latest/index.html).
