# Supplementary material for: The complex rupture evolution of the long and slow, tsunamigenic 2021 South Sandwich Islands earthquake
Source: Sci Rep. 2025 May 21;15:17706. doi: 10.1038/s41598-025-02043-6 (PMC12095499; doi:10.1038/s41598-025-02043-6)
Supplement: Supplementary file 1 — Supplementary Material 1 [file 41598_2025_2043_MOESM1_ESM.pdf]

Supporting Information for

The complex rupture evolution behind the long and slow, tsunamigenic 2021 South Sandwich Islands earthquake

Ryo Yamaguchi[1], Yuji Yagi[2], Ryo Okuwaki[2], and Bogdan Enescu[3,4]

[1]Graduate School of Science and Technology, University of Tsukuba, Tennodai 1-1-1, Tsukuba, Ibaraki 305– 8572, Japan

[2]Institute of Life and Environmental Sciences, University of Tsukuba, Tennodai 1-1-1, Tsukuba, Ibaraki 305– 8572, Japan

[3]Department of Geophysics, Graduate School of Science, Kyoto University, Kitashirakawa, Oiwake-cho, Sakyo-ku, Kyoto 606-8502, Japan

[4]National Institute for Earth Physics, Calugareni str. 12, P.O. Box MG-2, 077125, Magurele-Bucharest, Ilfov, Romania

Corresponding authors: Ryo Yamaguchi (yama0109@geol.tsukuba.ac.jp) and
Yuji Yagi (yagi-y@geol.tsukuba.ac.jp)

Contents

Tables S1 to **S3.** 1-D structure models used for calculating the Green's function.

Figure S1. Summary of the empirical Green's function analysis.

Figure S2. Comparison of observed waveforms with synthetic waveforms.

Figure S3. Full snapshots of potency-rate density evolution for Episode 1 and 2.

Figure S4. Full snapshots of potency-rate density evolution for Episode 3.

Figure S5. Full snapshots of potency-rate density evolution for Episode 4.

Figure S6. Comparison of the potency-rate density evolution projected along strike and sub-events identified by previous studies.

Figure S7. The dip angle distribution.

Table S1. 1-D structure around the source used for calculating the Green's function, based on CRUST1.0 model[1]

V_P (km/s)	V_S (km/s)	Density (10^3 kg/m^3)	Thickness (km)
1.50	0.00	1.02	3.80
5.75	3.20	2.70	3.20
7.10	4.05	3.05	4.80
8.04	4.47	3.32	- (below moho)

Table S2. 1-D structure around the source used for calculating the Green's function, based on CRUST2.0 model[2]

V_P (km/s)	V_S (km/s)	Density (10^3 kg/m^3)	Thickness (km)
1.50	0.00	1.02	3.61
5.00	2.50	2.60	1.70
6.60	3.65	2.90	2.30
7.10	3.90	3.05	2.50
8.15	4.65	3.35	- (below moho)

Table S3. 1-D structure around the source used for calculating the Green's function, based on ak135-F model[3,4]

V_P (km/s)	V_S (km/s)	Density (10^3 kg/m^3)	Thickness (km)
1.45	0.00	1.02	3.00
5.80	3.20	2.60	6.70
6.80	3.90	2.92	8.00
8.04	4.48	3.64	- (below moho)

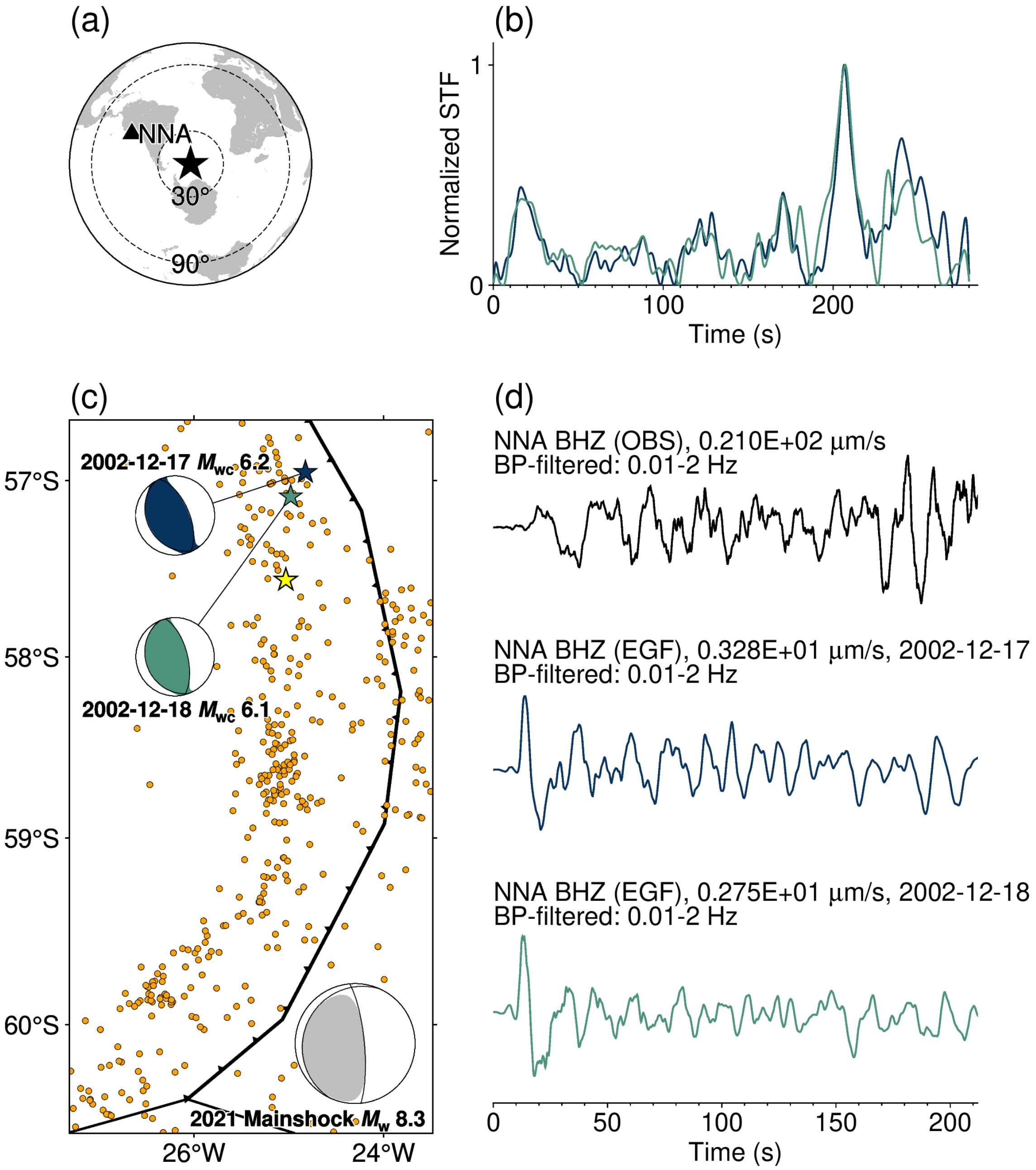

Figure S1. Summary of the empirical Green's function (EGF) analysis. (a) An azimuthal equidistant projection of the location of NNA station. (b) The normalized source time function obtained by using observed waveforms of the 2002-12-17 (blue) and the 2002-12-18 (green) event as EGFs. A band-pass filter of 0.01 to 2 Hz was applied to the observed waveform and EGFs. (c) Map projection of the GCMT[5,6] solution of the mainshock (grey beachball) and the USGS's[7] two events used as EGFs. The blue and green stars are the epicentres of the 2002-12-17 and 2002-12-18 event, respectively. The yellow star is the epicentre of the first event of the 2021 SSI earthquake. Orange dots are aftershocks that occurred within 3 days of the mainshock detected by the USGS. The black lines are the plate boundary[8]. (d) Observed waveform of the 2021 SSI earthquake (upper) and observed waveforms of the 2002-12-17 event (blue) and the 2002-12-18 event (green) used as EGFs. Both observed waveform and EGFs are band-pass (0.01–2 Hz) filtered. This figure was made with the Generic Mapping Tools (v 6.5.0) software[9].

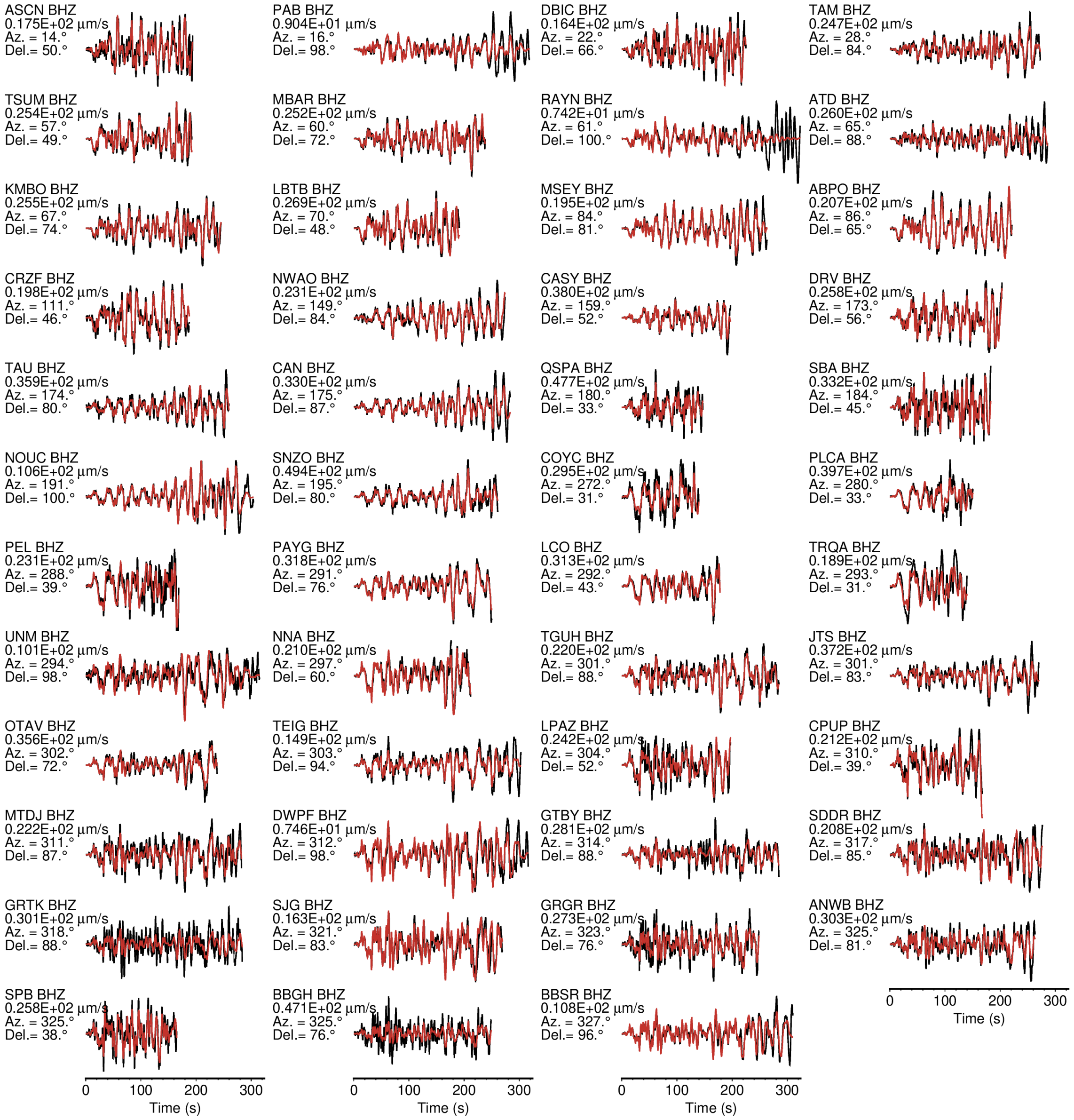

Figure S2. Comparison of observed waveforms (black) with synthetic waveforms (red). Each panel is labelled with the station name, maximum amplitude, azimuth (Az.), and epicentral distance (Del.) from the mainshock. These waveforms were resampled to 20 Hz for plotting. This figure was made with the Generic Mapping Tools (v 6.5.0) software[9].

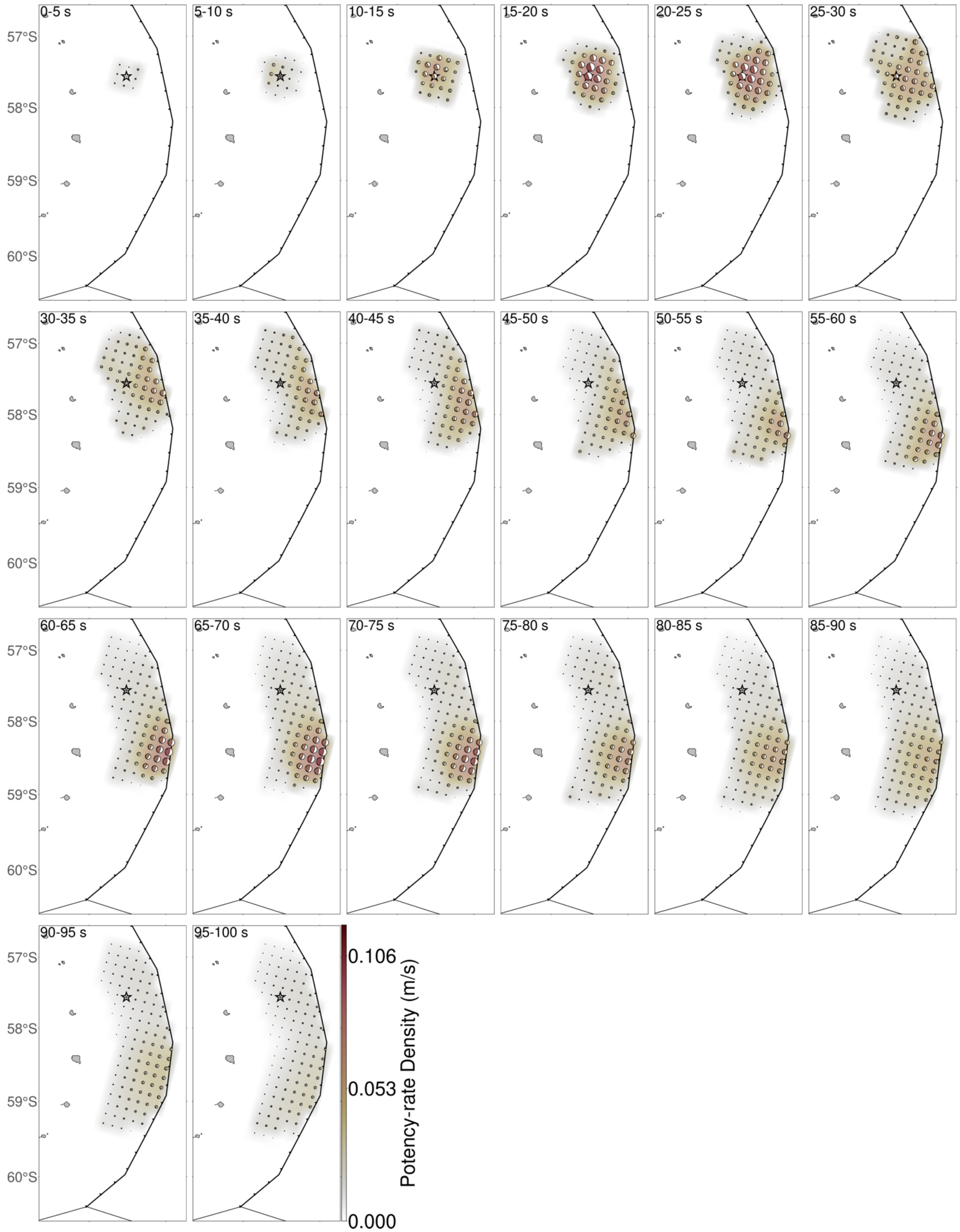

Figure S3. Full snapshots of potency-rate density evolution for Episodes 1 and 2. The beachballs show the potency-rate density tensors. This figure was made with the Generic Mapping Tools (v 6.5.0) software[9].

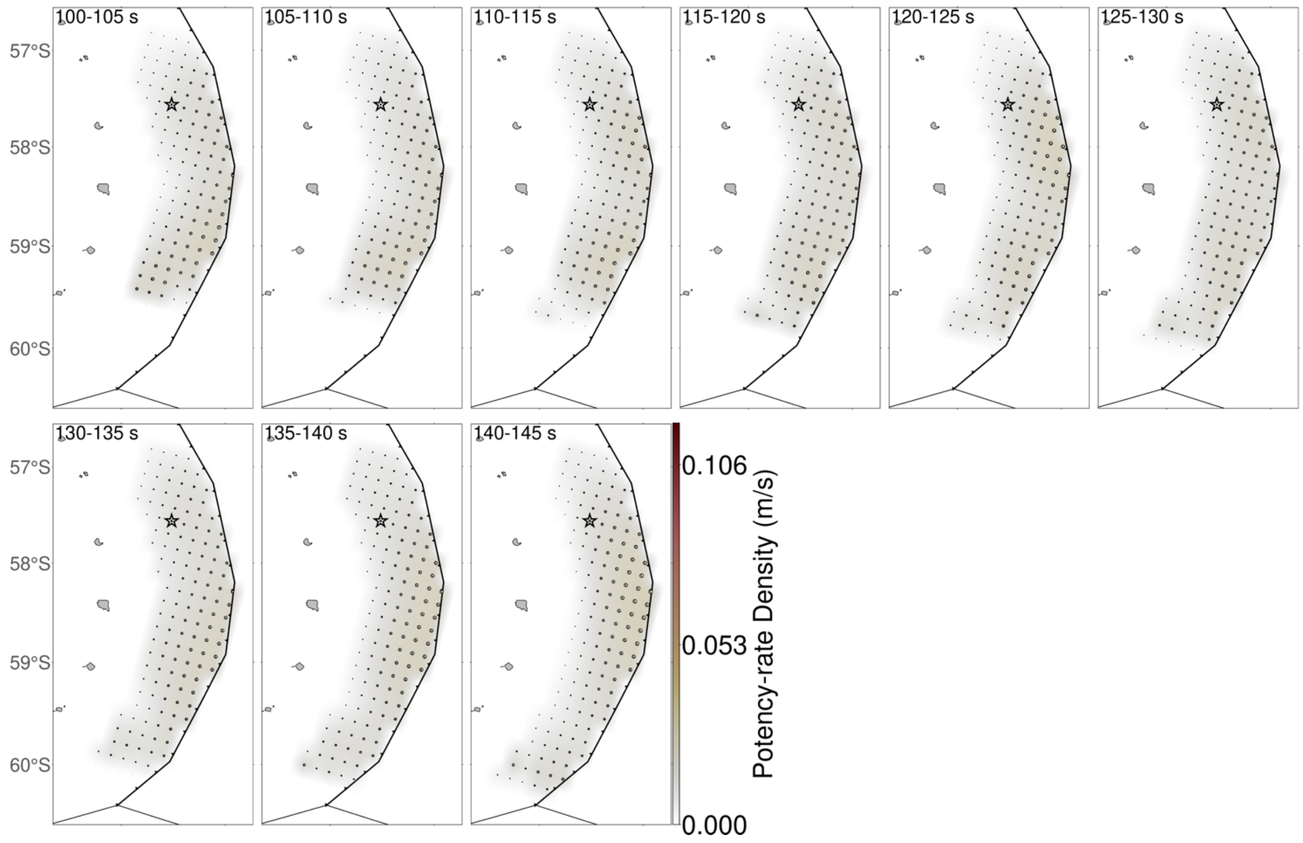

Figure S4. Full snapshots of potency-rate density evolution for Episode 3. The beachballs show the potency-rate density tensors. This figure was made with the Generic Mapping Tools (v 6.5.0) software[9].

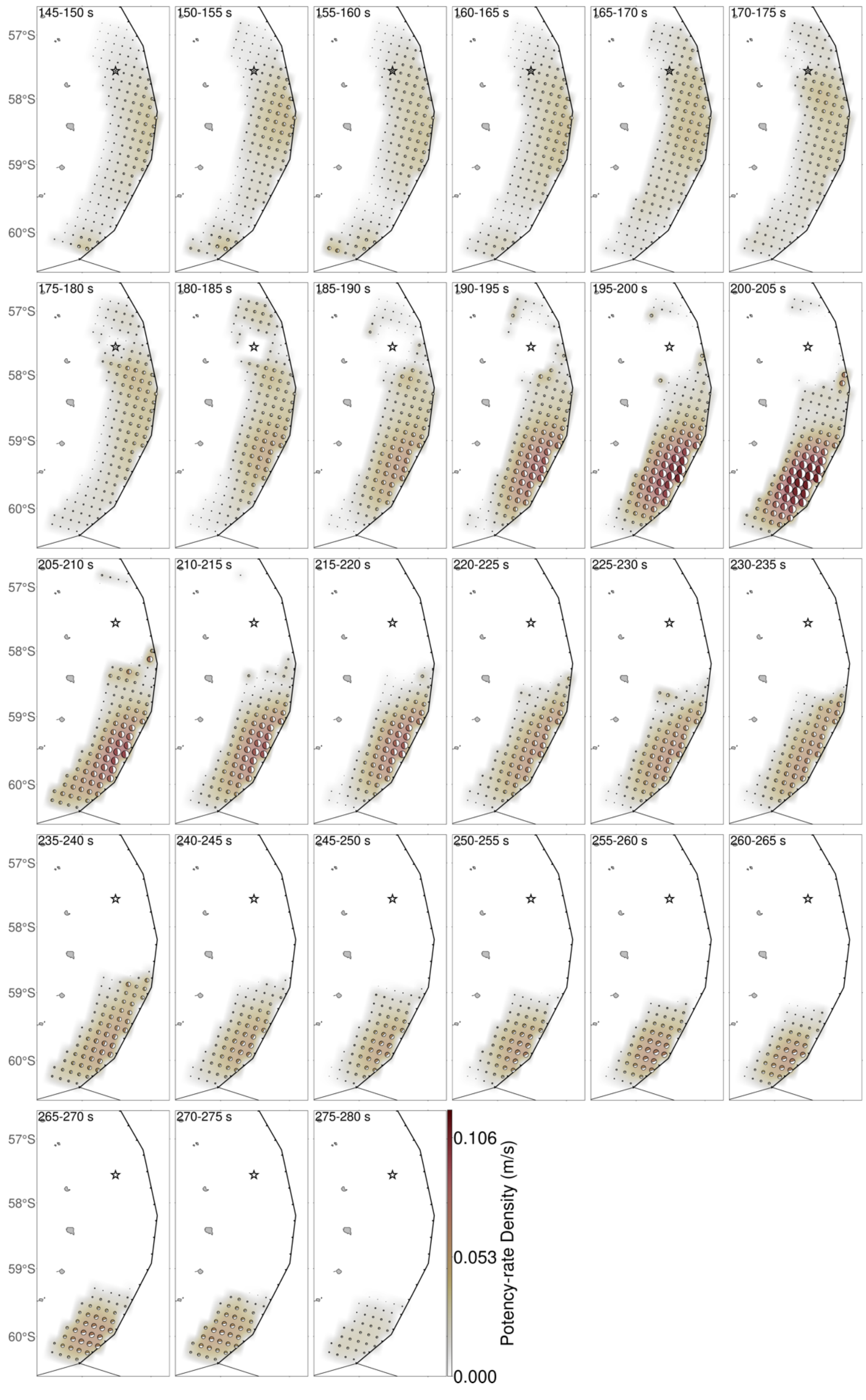

Figure S5. Full snapshots of potency-rate density evolution for Episode 4. The beachballs show the potency-rate density tensors. This figure was made with the Generic Mapping Tools (v 6.5.0) software[9].

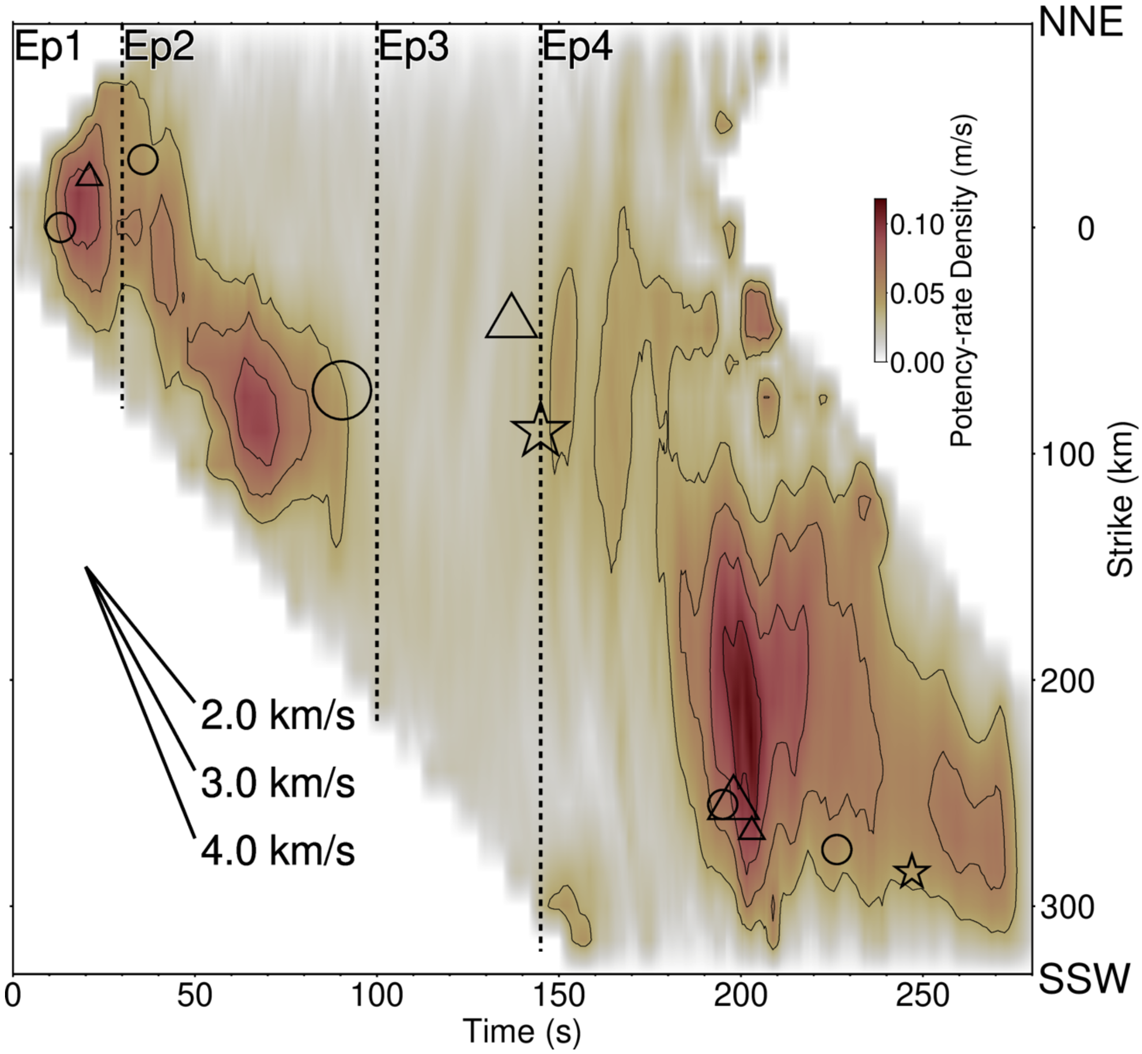

Figure S6

Comparison of the potency-rate density evolution projected along strike and sub-events identified by previous studies[10,11]. The circle indicates the centroid of the sub-events reported by Jia et al.[10], and the triangle indicates the location of the centroid moment tensor reported by Metz et al.[11] The larger markers are the events with duration longer than 100 s. The large and small stars are the epicentres of the M_W 8.1 and mb 6.7 events determined by the USGS[7], respectively. The style of contours is the same as drawn in Fig. 2d. This figure was made with the Generic Mapping Tools (v 6.5.0) software[9].

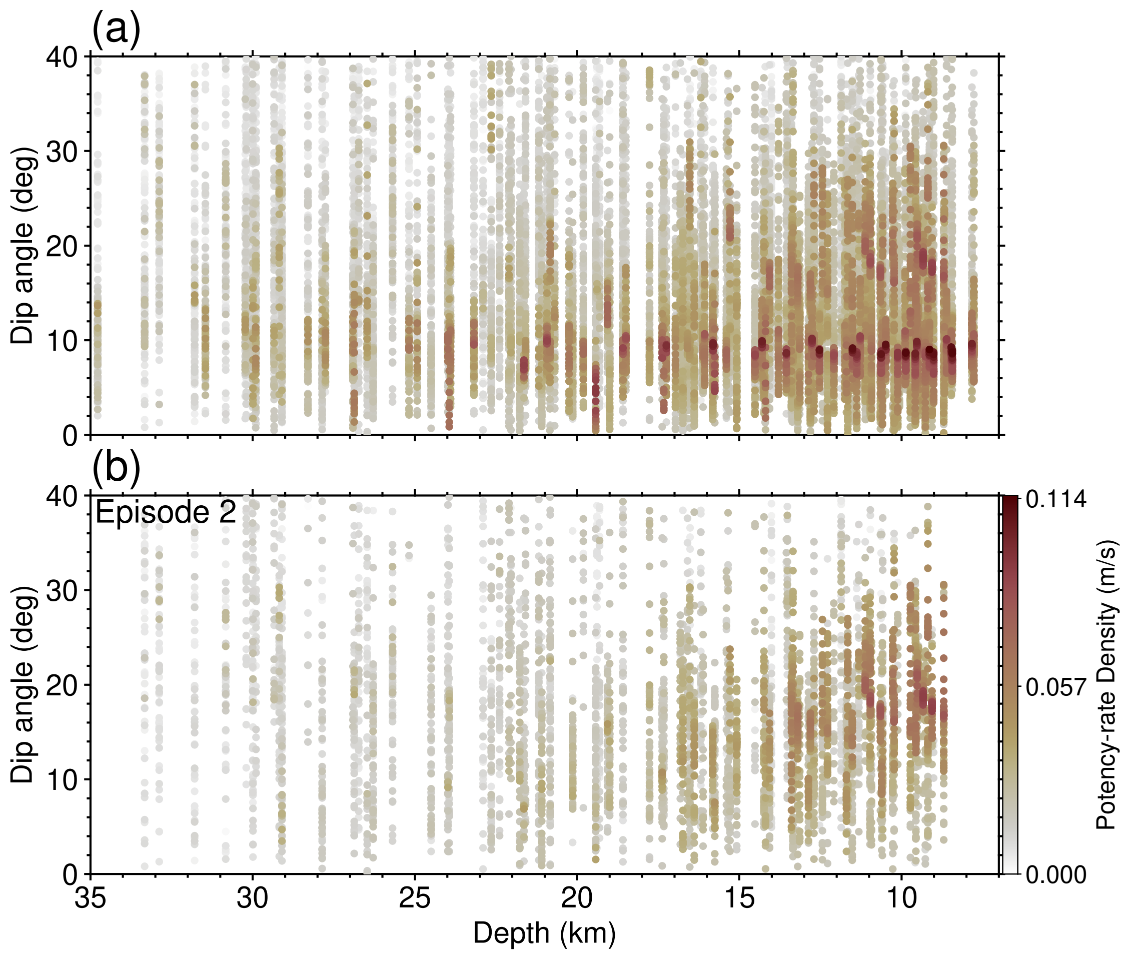

Figure S7

The dip angle distribution. The dip angle is derived from our best-fitting double couple solution of our potency-rate density tensor in a 1-s time window at each source knot in the model domain. The dip angle from the two possible nodal planes is selected based on the subducted plate surface. (a) The dip angle distribution of all the model space. (b) The dip angle distribution during OT+70–100 s in Episode 2.

Reference

1. Laske, G., Masters, G., Ma, Z. & Pasyanos, M. Update on CRUST1.0 - A 1-degree Global Model of Earth's Crust. EGU2013-2658 (2013).
2. Bassin, C., Laske, G. & Masters, G. The Current Limits of Resolution for Surface Wave Tomography in North America. *EOS Trans AGU* **81**, (2000).
3. Kennett, B. L. N., Engdahl, E. R. & Buland, R. Constraints on seismic velocities in the Earth from traveltimes. *Geophysical Journal International* **122**, 108–124 (1995).
4. Montagner, J.-P. & Kennett, B. L. N. How to reconcile body-wave and normal-mode reference earth models. *Geophysical Journal International* **125**, 229–248 (1996).
5. Dziewonski, A. M., Chou, T.-A. & Woodhouse, J. H. Determination of earthquake source parameters from waveform data for studies of global and regional seismicity. *J. Geophys. Res.* **86**, 2825–2852 (1981).
6. Ekström, G., Nettles, M. & Dziewoński, A. M. The global CMT project 2004–2010: Centroid-moment tensors for 13,017 earthquakes. *Physics of the Earth and Planetary Interiors* **200–201**, 1–9 (2012).
7. U. S. Geological Survey. Advanced National Seismic System (ANSS) Comprehensive Catalog. (2017) doi:10.5066/F7MS3QZH.
8. Bird, P. An updated digital model of plate boundaries: UPDATED MODEL OF PLATE BOUNDARIES. *Geochem. Geophys. Geosyst.* **4**, (2003).
9. Wessel, P. *et al.* The Generic Mapping Tools Version 6. *Geochem. Geophys. Geosyst.* **20**, 5556–5564 (2019).
10. Jia, Z., Zhan, Z. & Kanamori, H. The 2021 South Sandwich Island M_w 8.2 Earthquake: A Slow Event Sandwiched Between Regular Ruptures. *Geophysical Research Letters* **49**, (2022).
11. Metz, M. *et al.* Seismic and Tsunamigenic Characteristics of a Multimodal Rupture of Rapid and Slow Stages: The Example of the Complex 12 August 2021 South Sandwich Earthquake. *JGR Solid Earth* **127**, (2022).
